# 3-[(1-Hy­droxy-1-phenyl­propan-2-yl)amino]-5,5-dimethyl­cyclo­hex-2-enone

**DOI:** 10.1107/S160053681201570X

**Published:** 2012-04-18

**Authors:** Mostafa M. Ghorab, Mansour S. Al-Said, Saleh I. Alqasoumi, Tze Shyang Chia, Hoong-Kun Fun

**Affiliations:** a Medicinal, Aromatic and Poisonous Plants Research Center (MAPPRC), College of Pharmacy, King Saud University, PO Box 2457, Riyadh 11451, Saudi Arabia; b Department of Pharmacognosy, College of Pharmacy, King Saud University, Saudi Arabia; c X-ray Crystallography Unit, School of Physics, Universiti Sains Malaysia, 11800 USM, Penang, Malaysia

## Abstract

The asymmetric unit of the title compound, C_17_H_23_NO_2_, consists of two crystallographically independent mol­ecules (*A* and *B*). The cyclo­hexene rings in both mol­ecules adopt an envelope conformation. In the crystal, independent mol­ecules, *A* and *B*, are each linked by inter­molecular bifurcated (N,O)—H⋯O hydrogen bonds, generating *R*_2_^1^(7) ring motifs and forming infinite chains along the *b* axis.

## Related literature

For cyclo­hex-2-enone derivatives and their biological activity, see: Ghorab *et al.* (2009 ▶, 2010 ▶); Ghorab, Al-Said & El-Hossary (2011 ▶); Aghil *et al.* (1992 ▶); Li & Strobel (2001 ▶). For the biological activity of phenyl­propan-2-yl­amino, see: Zhang *et al.* (2011 ▶). For the synthesis of biologically active heterocyclic compounds, see: Ghorab *et al.* (2012 ▶); Ghorab, Ragab *et al.* (2011 ▶). For hydrogen-bond motifs, see: Bernstein *et al.* (1995 ▶). For ring conformations, see: Cremer & Pople (1975 ▶).
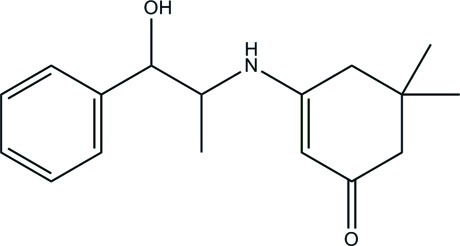


## Experimental

### 

#### Crystal data

C_17_H_23_NO_2_
*M* *_r_* = 273.36
Monoclinic, 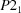

*a* = 10.4357 (6) Å
*b* = 12.4953 (8) Å
*c* = 12.8706 (5) Å
β = 107.019 (3)°
*V* = 1604.79 (15) Å^3^
*Z* = 4
Cu *K*α radiation
μ = 0.58 mm^−1^
*T* = 296 K
0.80 × 0.59 × 0.03 mm

#### Data collection

Bruker SMART APEXII CCD area-detector diffractometer
Absorption correction: multi-scan (*SADABS*; Bruker, 2009 ▶) *T*_min_ = 0.654, *T*_max_ = 0.983
8364 measured reflections
3114 independent reflections
2426 reflections with *I* > 2σ(*I*)
*R*_int_ = 0.051

#### Refinement

*R*[*F* ^2^ > 2σ(*F* ^2^)] = 0.052
*wR*(*F* ^2^) = 0.150
*S* = 1.08
3114 reflections
379 parameters
1 restraint
H atoms treated by a mixture of independent and constrained refinement
Δρ_max_ = 0.18 e Å^−3^
Δρ_min_ = −0.17 e Å^−3^

### 

Data collection: *APEX2* (Bruker, 2009 ▶); cell refinement: *SAINT* (Bruker, 2009 ▶); data reduction: *SAINT*; program(s) used to solve structure: *SHELXTL* (Sheldrick, 2008 ▶); program(s) used to refine structure: *SHELXTL*; molecular graphics: *SHELXTL*; software used to prepare material for publication: *SHELXTL* and *PLATON* (Spek, 2009 ▶).

## Supplementary Material

Crystal structure: contains datablock(s) global, I. DOI: 10.1107/S160053681201570X/is5115sup1.cif

Structure factors: contains datablock(s) I. DOI: 10.1107/S160053681201570X/is5115Isup2.hkl

Supplementary material file. DOI: 10.1107/S160053681201570X/is5115Isup3.cml

Additional supplementary materials:  crystallographic information; 3D view; checkCIF report

## Figures and Tables

**Table 1:** Hydrogen-bond geometry (Å, °)

*D*—H⋯*A*	*D*—H	H⋯*A*	*D*⋯*A*	*D*—H⋯*A*
N1*B*—H1*NB*⋯O1*B*^i^	0.82 (6)	2.12 (6)	2.874 (4)	155 (5)
O2*B*—H1*OB*⋯O1*B*^i^	0.96 (7)	1.75 (7)	2.701 (4)	170 (6)
N1*A*—H1*NA*⋯O1*A*^ii^	0.85 (6)	2.04 (6)	2.853 (4)	160 (5)
O2*A*—H1*OA*⋯O1*A*^ii^	0.91 (7)	1.88 (7)	2.724 (4)	155 (6)

Symmetry codes: (i) 
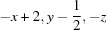
; (ii) 
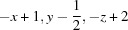
.

**Fig. 1.**
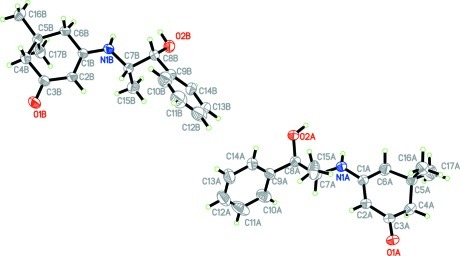
The molecular structure of the title compound with atom labels with 30% probability displacement ellipsoids.

**Fig. 2.**
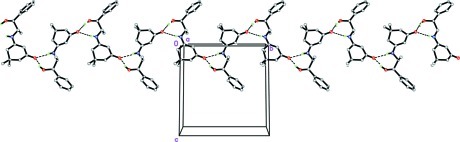
A part of crystal packing of the title compound. The dashed lines represent the hydrogen bonds.

## supplementary crystallographic information

### Comment

From literature survey it was found that cyclohex-2-enone derivatives are useful in the synthesis of heterocyclic compounds, especially quinoline derivatives (Ghorab *et al.*, 2009, 2010; Ghorab, Al-Said & El-Hossary, 2011). Cyclohex-2-enone derivatives also exhibit a wide range of biological activities such as anticancer (Aghil *et al.*, 1992) and antimicrobial (Li & Strobel, 2001) activities. On the other hand, compounds having the phenylpropan-2-ylamino moiety are also known to possess a wide range of biological and pharmacological activities, especially anticancer activity (Zhang *et al.*, 2011). In the light of these facts and as a continuation of our efforts towards synthesizing biologically active heterocyclic compounds (Ghorab, Ragab *et al.*, 2011; Ghorab *et al.*, 2012), we prepared a novel cyclohex-2-enone carrying a biologically active phenylpropan-2-ylamino moiety to evaluate its anticancer activity.

The asymmetric unit of the title compound consists of two crystallographically independent molecules (*A* and *B*) as shown in Fig. 1. In both molecules, the cyclohexene rings adopt an envelope conformation with puckering parameters (Cremer & Pople, 1975), *Q* = 0.436 (5) Å, θ = 128.6 (7)° and φ = 45.0 (8)° in molecule *A* [*Q* = 0.448 (4) Å, θ = 124.1 (5)° and φ = 54.4 (6)° in molecule *B*]. The distance of atom C5 from the mean plane of C1–C4/C6 is 0.5989 (68) Å in molecule *A*, whereas in molecule *B*, the corresponding distance is 0.6264 (51) Å. In molecule *A*, the mean plane of O1/C1–C4/C6 [maximum deviation = 0.0704 (30) Å at atom C6] forms dihedral angle of 61.13 (18)° with the terminal C9–C14 benzene ring, whereas in molecule *B*, the corresponding maximum deviation and dihedral angle are 0.0261 (27) Å at atom C1 and 56.20 (16)°, respectively.

In the crystal (Fig. 2), molecules are linked by intermolecular bifurcated N1A—H1NA···O1A, N1B—H1NB···O1B, O2A—H1OA···O1A and O2B—H1OB···O1B hydrogen bonds (Table 1), generating *R*_2_^1^(7) ring motifs (Bernstein *et al.*, 1995) and forming infinite chains along the *b* axis.

### Experimental

A mixture of 5,5-dimethylcyclohexane-1,3-dione (1.4 g, 0.01 mole) and 2-amino-1-phenylpropan-1-ol (1.51 g, 0.01 mole) in dry dimethylformamide (10 ml) containing triethylamine (3 drops) as catalyst was refluxed for 8 h. The obtained solid was recrystallized from ethanol to give the title compound. Single crystals which are suitable for an X-ray structural analysis were obtained by slow evaporation from ethanol at room temperature.

### Refinement

Atoms H1NA, H1NB, H1OA and H1OB were located from difference fourier map and refined using a riding model with *U*_iso_(H) = 1.5*U*_eq_(N or O), [N—H = 0.82 (6) and 0.85 (6) Å; O—H = 0.96 (6) and 0.91 (7) Å]. The remaining H atoms were positioned geometrically (C—H = 0.93, 0.96, 0.97 and 0.98 Å) and refined using a riding model with *U*_iso_(H) = 1.2 or 1.5*U*_eq_(C). A rotating group model was applied to the methyl groups. An outliner (1 0 0) was omitted. The absolute configuration cannot be determined because the anomalous dispersions are insufficient although Cu radiation was used. The crystal is not an inversion twin. In the final refinement, 1395 Friedel pairs were merged.

### Crystal data

**Table:**

C_17_H_23_NO_2_	*F*(000) = 592
*M**_r_* = 273.36	*D*_x_ = 1.131 Mg m^−^^3^
Monoclinic, *P*2_1_	Cu *K*α radiation, λ = 1.54178 Å
Hall symbol: P 2yb	Cell parameters from 877 reflections
*a* = 10.4357 (6) Å	θ = 3.6–67.1°
*b* = 12.4953 (8) Å	µ = 0.58 mm^−^^1^
*c* = 12.8706 (5) Å	*T* = 296 K
β = 107.019 (3)°	Plate, colourless
*V* = 1604.79 (15) Å^3^	0.80 × 0.59 × 0.03 mm
*Z* = 4	

### Data collection

**Table:**

Bruker SMART APEXII CCD area-detector diffractometer	3114 independent reflections
Radiation source: fine-focus sealed tube	2426 reflections with *I* > 2σ(*I*)
Graphite monochromator	*R*_int_ = 0.051
φ and ω scans	θ_max_ = 70.0°, θ_min_ = 3.6°
Absorption correction: multi-scan (*SADABS*; Bruker, 2009)	*h* = −12→12
*T*_min_ = 0.654, *T*_max_ = 0.983	*k* = −14→13
8364 measured reflections	*l* = −15→14

### Refinement

**Table:**

Refinement on *F*^2^	Primary atom site location: structure-invariant direct methods
Least-squares matrix: full	Secondary atom site location: difference Fourier map
*R*[*F*^2^ > 2σ(*F*^2^)] = 0.052	Hydrogen site location: inferred from neighbouring sites
*wR*(*F*^2^) = 0.150	H atoms treated by a mixture of independent and constrained refinement
*S* = 1.08	*w* = 1/[σ^2^(*F*_o_^2^) + (0.0759*P*)^2^ + 0.0565*P*] where *P* = (*F*_o_^2^ + 2*F*_c_^2^)/3
3114 reflections	(Δ/σ)_max_ < 0.001
379 parameters	Δρ_max_ = 0.18 e Å^−^^3^
1 restraint	Δρ_min_ = −0.17 e Å^−^^3^

### Special details

**Table:**

Geometry. All e.s.d.'s (except the e.s.d. in the dihedral angle between two l.s. planes) are estimated using the full covariance matrix. The cell e.s.d.'s are taken into account individually in the estimation of e.s.d.'s in distances, angles and torsion angles; correlations between e.s.d.'s in cell parameters are only used when they are defined by crystal symmetry. An approximate (isotropic) treatment of cell e.s.d.'s is used for estimating e.s.d.'s involving l.s. planes.
Refinement. Refinement of *F*^2^ against ALL reflections. The weighted *R*-factor *wR* and goodness of fit *S* are based on *F*^2^, conventional *R*-factors *R* are based on *F*, with *F* set to zero for negative *F*^2^. The threshold expression of *F*^2^ > σ(*F*^2^) is used only for calculating *R*-factors(gt) *etc*. and is not relevant to the choice of reflections for refinement. *R*-factors based on *F*^2^ are statistically about twice as large as those based on *F*, and *R*- factors based on ALL data will be even larger.

### Fractional atomic coordinates and isotropic or equivalent isotropic displacement parameters (Å^2^)

**Table:**

	*x*	*y*	*z*	*U*_iso_*/*U*_eq_	
N1B	0.9656 (3)	0.7034 (2)	0.0647 (2)	0.0564 (7)	
H1NB	0.952 (5)	0.639 (5)	0.063 (4)	0.085*	
O1B	1.0743 (4)	0.9891 (2)	−0.1303 (2)	0.0837 (9)	
O2B	0.8674 (3)	0.6373 (2)	0.2624 (2)	0.0846 (9)	
H1OB	0.878 (6)	0.582 (6)	0.214 (5)	0.127*	
C1B	0.9999 (4)	0.7436 (2)	−0.0189 (2)	0.0520 (7)	
C2B	1.0122 (4)	0.8512 (3)	−0.0350 (3)	0.0606 (9)	
H2BA	0.9890	0.8993	0.0116	0.073*	
C3B	1.0586 (4)	0.8914 (3)	−0.1194 (3)	0.0594 (8)	
C4B	1.0952 (4)	0.8145 (3)	−0.1963 (3)	0.0619 (8)	
H4BA	1.1904	0.7991	−0.1696	0.074*	
H4BB	1.0785	0.8486	−0.2668	0.074*	
C5B	1.0176 (3)	0.7093 (3)	−0.2104 (2)	0.0542 (7)	
C6B	1.0283 (4)	0.6640 (3)	−0.0971 (3)	0.0591 (8)	
H6BA	0.9662	0.6047	−0.1051	0.071*	
H6BB	1.1180	0.6358	−0.0659	0.071*	
C7B	0.9421 (4)	0.7699 (3)	0.1507 (2)	0.0562 (8)	
H7BA	1.0048	0.8301	0.1619	0.067*	
C8B	0.9730 (4)	0.7088 (3)	0.2587 (3)	0.0586 (8)	
H8BA	1.0552	0.6671	0.2680	0.070*	
C9B	0.9958 (4)	0.7881 (3)	0.3519 (2)	0.0629 (9)	
C10B	1.1185 (5)	0.8390 (4)	0.3883 (3)	0.0864 (12)	
H10A	1.1868	0.8201	0.3589	0.104*	
C11B	1.1415 (7)	0.9165 (5)	0.4665 (4)	0.117 (2)	
H11A	1.2244	0.9504	0.4891	0.141*	
C12B	1.0439 (9)	0.9441 (4)	0.5110 (4)	0.123 (3)	
H12A	1.0599	0.9967	0.5644	0.147*	
C13B	0.9188 (8)	0.8938 (4)	0.4771 (4)	0.1056 (19)	
H13A	0.8515	0.9132	0.5073	0.127*	
C14B	0.8957 (5)	0.8144 (3)	0.3974 (3)	0.0809 (12)	
H14A	0.8135	0.7795	0.3752	0.097*	
C15B	0.8015 (5)	0.8165 (4)	0.1162 (3)	0.0850 (12)	
H15A	0.7845	0.8488	0.0458	0.127*	
H15B	0.7936	0.8696	0.1679	0.127*	
H15C	0.7375	0.7605	0.1131	0.127*	
C16B	1.0812 (5)	0.6296 (4)	−0.2715 (4)	0.0860 (13)	
H16A	1.0724	0.6562	−0.3432	0.129*	
H16B	1.0366	0.5618	−0.2765	0.129*	
H16C	1.1744	0.6209	−0.2330	0.129*	
C17B	0.8726 (4)	0.7259 (4)	−0.2740 (3)	0.0769 (11)	
H17A	0.8673	0.7534	−0.3447	0.115*	
H17B	0.8323	0.7760	−0.2364	0.115*	
H17C	0.8258	0.6589	−0.2813	0.115*	
N1A	0.4987 (3)	0.9812 (3)	0.9032 (2)	0.0639 (8)	
H1NA	0.475 (5)	0.916 (5)	0.895 (4)	0.096*	
O1A	0.5349 (4)	1.2606 (2)	1.1557 (2)	0.0891 (10)	
O2A	0.5582 (4)	0.8861 (2)	0.7091 (2)	0.0911 (10)	
H1OA	0.544 (6)	0.830 (6)	0.749 (5)	0.137*	
C1A	0.4810 (4)	1.0239 (3)	0.9930 (3)	0.0608 (8)	
C2A	0.5195 (4)	1.1260 (3)	1.0280 (3)	0.0654 (9)	
H2AA	0.5631	1.1675	0.9887	0.078*	
C3A	0.4953 (5)	1.1699 (3)	1.1210 (3)	0.0691 (10)	
C4A	0.4101 (5)	1.1066 (3)	1.1752 (4)	0.0796 (12)	
H4AA	0.3167	1.1235	1.1404	0.095*	
H4AB	0.4313	1.1287	1.2506	0.095*	
C5A	0.4293 (4)	0.9850 (3)	1.1710 (3)	0.0636 (9)	
C6A	0.4110 (5)	0.9530 (3)	1.0537 (3)	0.0801 (11)	
H6AA	0.4436	0.8804	1.0526	0.096*	
H6AB	0.3159	0.9528	1.0154	0.096*	
C7A	0.5487 (4)	1.0387 (3)	0.8248 (3)	0.0602 (8)	
H7AA	0.5130	1.1117	0.8191	0.072*	
C8A	0.4965 (4)	0.9862 (3)	0.7130 (3)	0.0634 (9)	
H8AA	0.3997	0.9753	0.6968	0.076*	
C9A	0.5223 (4)	1.0579 (3)	0.6266 (3)	0.0666 (10)	
C10A	0.4390 (6)	1.1427 (4)	0.5867 (4)	0.0984 (14)	
H10B	0.3629	1.1536	0.6090	0.118*	
C11A	0.4696 (9)	1.2140 (5)	0.5108 (5)	0.123 (2)	
H11B	0.4134	1.2715	0.4832	0.148*	
C12A	0.5814 (8)	1.1977 (5)	0.4787 (4)	0.110 (2)	
H12B	0.6027	1.2451	0.4306	0.132*	
C13A	0.6602 (7)	1.1139 (5)	0.5162 (4)	0.1065 (17)	
H13B	0.7352	1.1023	0.4927	0.128*	
C14A	0.6318 (6)	1.0442 (4)	0.5895 (3)	0.0844 (12)	
H14B	0.6884	0.9862	0.6145	0.101*	
C15A	0.7007 (5)	1.0462 (6)	0.8609 (3)	0.0994 (17)	
H15E	0.7317	1.0599	0.9376	0.149*	
H15F	0.7285	1.1034	0.8226	0.149*	
H15G	0.7379	0.9800	0.8453	0.149*	
C16A	0.5681 (5)	0.9551 (4)	1.2415 (4)	0.0882 (12)	
H16E	0.5811	0.8794	1.2367	0.132*	
H16F	0.5771	0.9740	1.3156	0.132*	
H16G	0.6340	0.9928	1.2171	0.132*	
C17A	0.3260 (6)	0.9282 (5)	1.2138 (4)	0.1088 (17)	
H17E	0.3320	0.8524	1.2042	0.163*	
H17F	0.2378	0.9525	1.1746	0.163*	
H17D	0.3429	0.9439	1.2896	0.163*	

### Atomic displacement parameters (Å^2^)

**Table:**

	*U*^11^	*U*^22^	*U*^33^	*U*^12^	*U*^13^	*U*^23^
N1B	0.084 (2)	0.0409 (13)	0.0474 (13)	0.0020 (14)	0.0233 (13)	0.0002 (11)
O1B	0.140 (3)	0.0443 (14)	0.0780 (16)	−0.0080 (15)	0.0496 (17)	0.0045 (12)
O2B	0.138 (3)	0.0543 (15)	0.0801 (17)	−0.0153 (16)	0.0614 (18)	−0.0080 (13)
C1B	0.071 (2)	0.0410 (15)	0.0446 (14)	0.0053 (15)	0.0182 (14)	0.0010 (13)
C2B	0.094 (3)	0.0399 (16)	0.0526 (15)	0.0081 (16)	0.0291 (17)	−0.0021 (13)
C3B	0.081 (2)	0.0441 (17)	0.0542 (16)	−0.0001 (16)	0.0215 (16)	0.0040 (14)
C4B	0.078 (2)	0.0589 (19)	0.0542 (16)	−0.0002 (18)	0.0268 (15)	0.0031 (15)
C5B	0.070 (2)	0.0502 (16)	0.0457 (14)	0.0033 (16)	0.0220 (14)	−0.0029 (13)
C6B	0.082 (2)	0.0437 (16)	0.0536 (16)	0.0120 (16)	0.0234 (16)	0.0020 (14)
C7B	0.077 (2)	0.0489 (17)	0.0471 (15)	0.0067 (16)	0.0255 (15)	0.0002 (13)
C8B	0.078 (2)	0.0496 (17)	0.0538 (16)	0.0051 (17)	0.0286 (15)	0.0057 (14)
C9B	0.093 (3)	0.0532 (18)	0.0451 (15)	0.0101 (19)	0.0241 (16)	0.0061 (14)
C10B	0.094 (3)	0.093 (3)	0.065 (2)	−0.004 (3)	0.012 (2)	−0.002 (2)
C11B	0.166 (6)	0.101 (4)	0.065 (3)	−0.023 (4)	0.004 (3)	−0.012 (3)
C12B	0.228 (8)	0.069 (3)	0.051 (2)	−0.004 (4)	0.010 (3)	−0.006 (2)
C13B	0.191 (6)	0.075 (3)	0.072 (3)	0.026 (4)	0.071 (3)	0.001 (2)
C14B	0.125 (3)	0.064 (2)	0.070 (2)	0.000 (2)	0.054 (2)	−0.0018 (18)
C15B	0.095 (3)	0.093 (3)	0.070 (2)	0.030 (3)	0.029 (2)	0.010 (2)
C16B	0.123 (4)	0.075 (3)	0.075 (2)	0.000 (3)	0.053 (2)	−0.015 (2)
C17B	0.085 (3)	0.083 (3)	0.0574 (18)	−0.011 (2)	0.0126 (17)	0.0062 (19)
N1A	0.093 (2)	0.0469 (15)	0.0562 (14)	−0.0041 (15)	0.0290 (15)	−0.0062 (13)
O1A	0.155 (3)	0.0436 (14)	0.0788 (16)	0.0037 (16)	0.0493 (18)	−0.0087 (12)
O2A	0.169 (3)	0.0426 (13)	0.0803 (17)	0.0055 (16)	0.066 (2)	0.0000 (12)
C1A	0.081 (2)	0.0493 (18)	0.0541 (16)	−0.0017 (17)	0.0226 (16)	−0.0055 (15)
C2A	0.097 (3)	0.0447 (17)	0.0619 (19)	−0.0023 (18)	0.0343 (18)	−0.0022 (14)
C3A	0.103 (3)	0.0435 (18)	0.0631 (19)	0.0125 (18)	0.0273 (19)	−0.0019 (15)
C4A	0.099 (3)	0.073 (3)	0.076 (2)	0.011 (2)	0.041 (2)	−0.0087 (19)
C5A	0.072 (2)	0.060 (2)	0.0662 (19)	−0.0035 (18)	0.0331 (18)	−0.0025 (16)
C6A	0.105 (3)	0.067 (2)	0.077 (2)	−0.021 (2)	0.041 (2)	−0.012 (2)
C7A	0.082 (2)	0.0480 (18)	0.0529 (16)	−0.0019 (17)	0.0239 (16)	−0.0034 (14)
C8A	0.086 (2)	0.0529 (18)	0.0518 (16)	−0.0053 (18)	0.0214 (16)	−0.0061 (15)
C9A	0.095 (3)	0.0534 (19)	0.0435 (14)	−0.0072 (19)	0.0079 (16)	−0.0027 (14)
C10A	0.106 (3)	0.083 (3)	0.086 (3)	0.012 (3)	−0.002 (3)	0.018 (3)
C11A	0.144 (6)	0.081 (3)	0.104 (4)	0.000 (4)	−0.027 (4)	0.032 (3)
C12A	0.161 (6)	0.085 (4)	0.070 (3)	−0.027 (4)	0.012 (3)	0.018 (2)
C13A	0.164 (5)	0.088 (3)	0.077 (3)	−0.025 (4)	0.049 (3)	0.005 (3)
C14A	0.132 (4)	0.067 (2)	0.0611 (19)	−0.001 (2)	0.040 (2)	0.0031 (18)
C15A	0.084 (3)	0.155 (5)	0.0532 (18)	−0.022 (3)	0.0101 (18)	−0.007 (3)
C16A	0.104 (3)	0.080 (3)	0.087 (3)	0.009 (3)	0.037 (2)	0.012 (2)
C17A	0.126 (4)	0.115 (4)	0.107 (3)	−0.027 (4)	0.066 (3)	−0.010 (3)

### Geometric parameters (Å, º)

**Table:**

N1B—C1B	1.327 (4)	N1A—C1A	1.334 (4)
N1B—C7B	1.461 (4)	N1A—C7A	1.454 (4)
N1B—H1NB	0.82 (6)	N1A—H1NA	0.85 (6)
O1B—C3B	1.245 (4)	O1A—C3A	1.245 (5)
O2B—C8B	1.430 (5)	O2A—C8A	1.414 (5)
O2B—H1OB	0.96 (6)	O2A—H1OA	0.91 (7)
C1B—C2B	1.373 (5)	C1A—C2A	1.374 (5)
C1B—C6B	1.505 (4)	C1A—C6A	1.504 (5)
C2B—C3B	1.406 (4)	C2A—C3A	1.405 (5)
C2B—H2BA	0.9300	C2A—H2AA	0.9300
C3B—C4B	1.506 (5)	C3A—C4A	1.505 (6)
C4B—C5B	1.527 (5)	C4A—C5A	1.536 (6)
C4B—H4BA	0.9700	C4A—H4AA	0.9700
C4B—H4BB	0.9700	C4A—H4AB	0.9700
C5B—C17B	1.510 (6)	C5A—C16A	1.514 (6)
C5B—C16B	1.536 (5)	C5A—C6A	1.519 (5)
C5B—C6B	1.538 (4)	C5A—C17A	1.522 (6)
C6B—H6BA	0.9700	C6A—H6AA	0.9700
C6B—H6BB	0.9700	C6A—H6AB	0.9700
C7B—C15B	1.519 (6)	C7A—C15A	1.519 (6)
C7B—C8B	1.535 (4)	C7A—C8A	1.530 (4)
C7B—H7BA	0.9800	C7A—H7AA	0.9800
C8B—C9B	1.520 (5)	C8A—C9A	1.512 (5)
C8B—H8BA	0.9800	C8A—H8AA	0.9800
C9B—C14B	1.378 (5)	C9A—C10A	1.371 (7)
C9B—C10B	1.383 (6)	C9A—C14A	1.372 (6)
C10B—C11B	1.367 (7)	C10A—C11A	1.426 (9)
C10B—H10A	0.9300	C10A—H10B	0.9300
C11B—C12B	1.351 (10)	C11A—C12A	1.362 (10)
C11B—H11A	0.9300	C11A—H11B	0.9300
C12B—C13B	1.398 (9)	C12A—C13A	1.331 (9)
C12B—H12A	0.9300	C12A—H12B	0.9300
C13B—C14B	1.397 (7)	C13A—C14A	1.379 (6)
C13B—H13A	0.9300	C13A—H13B	0.9300
C14B—H14A	0.9300	C14A—H14B	0.9300
C15B—H15A	0.9600	C15A—H15E	0.9600
C15B—H15B	0.9600	C15A—H15F	0.9600
C15B—H15C	0.9600	C15A—H15G	0.9600
C16B—H16A	0.9600	C16A—H16E	0.9600
C16B—H16B	0.9600	C16A—H16F	0.9600
C16B—H16C	0.9600	C16A—H16G	0.9600
C17B—H17A	0.9600	C17A—H17E	0.9600
C17B—H17B	0.9600	C17A—H17F	0.9600
C17B—H17C	0.9600	C17A—H17D	0.9600
			
C1B—N1B—C7B	123.0 (3)	C1A—N1A—C7A	125.0 (3)
C1B—N1B—H1NB	116 (3)	C1A—N1A—H1NA	112 (3)
C7B—N1B—H1NB	121 (3)	C7A—N1A—H1NA	123 (3)
C8B—O2B—H1OB	101 (4)	C8A—O2A—H1OA	121 (4)
N1B—C1B—C2B	123.5 (3)	N1A—C1A—C2A	123.6 (3)
N1B—C1B—C6B	116.4 (3)	N1A—C1A—C6A	115.2 (3)
C2B—C1B—C6B	120.1 (3)	C2A—C1A—C6A	121.1 (3)
C1B—C2B—C3B	122.4 (3)	C1A—C2A—C3A	122.2 (3)
C1B—C2B—H2BA	118.8	C1A—C2A—H2AA	118.9
C3B—C2B—H2BA	118.8	C3A—C2A—H2AA	118.9
O1B—C3B—C2B	121.6 (3)	O1A—C3A—C2A	122.6 (4)
O1B—C3B—C4B	119.0 (3)	O1A—C3A—C4A	119.4 (3)
C2B—C3B—C4B	119.4 (3)	C2A—C3A—C4A	117.9 (3)
C3B—C4B—C5B	113.5 (3)	C3A—C4A—C5A	113.7 (3)
C3B—C4B—H4BA	108.9	C3A—C4A—H4AA	108.8
C5B—C4B—H4BA	108.9	C5A—C4A—H4AA	108.8
C3B—C4B—H4BB	108.9	C3A—C4A—H4AB	108.8
C5B—C4B—H4BB	108.9	C5A—C4A—H4AB	108.8
H4BA—C4B—H4BB	107.7	H4AA—C4A—H4AB	107.7
C17B—C5B—C4B	111.0 (3)	C16A—C5A—C6A	110.6 (4)
C17B—C5B—C16B	109.0 (3)	C16A—C5A—C17A	108.8 (4)
C4B—C5B—C16B	109.0 (3)	C6A—C5A—C17A	109.9 (4)
C17B—C5B—C6B	110.4 (3)	C16A—C5A—C4A	109.5 (4)
C4B—C5B—C6B	108.4 (3)	C6A—C5A—C4A	108.4 (3)
C16B—C5B—C6B	108.9 (3)	C17A—C5A—C4A	109.6 (4)
C1B—C6B—C5B	114.6 (3)	C1A—C6A—C5A	115.0 (3)
C1B—C6B—H6BA	108.6	C1A—C6A—H6AA	108.5
C5B—C6B—H6BA	108.6	C5A—C6A—H6AA	108.5
C1B—C6B—H6BB	108.6	C1A—C6A—H6AB	108.5
C5B—C6B—H6BB	108.6	C5A—C6A—H6AB	108.5
H6BA—C6B—H6BB	107.6	H6AA—C6A—H6AB	107.5
N1B—C7B—C15B	111.0 (3)	N1A—C7A—C15A	111.9 (3)
N1B—C7B—C8B	111.5 (3)	N1A—C7A—C8A	109.8 (3)
C15B—C7B—C8B	112.6 (3)	C15A—C7A—C8A	111.5 (3)
N1B—C7B—H7BA	107.2	N1A—C7A—H7AA	107.8
C15B—C7B—H7BA	107.2	C15A—C7A—H7AA	107.8
C8B—C7B—H7BA	107.2	C8A—C7A—H7AA	107.8
O2B—C8B—C9B	108.9 (3)	O2A—C8A—C9A	108.5 (3)
O2B—C8B—C7B	112.4 (3)	O2A—C8A—C7A	111.9 (3)
C9B—C8B—C7B	109.4 (3)	C9A—C8A—C7A	110.2 (3)
O2B—C8B—H8BA	108.7	O2A—C8A—H8AA	108.7
C9B—C8B—H8BA	108.7	C9A—C8A—H8AA	108.7
C7B—C8B—H8BA	108.7	C7A—C8A—H8AA	108.7
C14B—C9B—C10B	119.2 (4)	C10A—C9A—C14A	117.9 (4)
C14B—C9B—C8B	121.8 (4)	C10A—C9A—C8A	120.3 (4)
C10B—C9B—C8B	118.8 (3)	C14A—C9A—C8A	121.8 (4)
C11B—C10B—C9B	121.4 (5)	C9A—C10A—C11A	119.6 (6)
C11B—C10B—H10A	119.3	C9A—C10A—H10B	120.2
C9B—C10B—H10A	119.3	C11A—C10A—H10B	120.2
C12B—C11B—C10B	120.1 (6)	C12A—C11A—C10A	119.9 (6)
C12B—C11B—H11A	119.9	C12A—C11A—H11B	120.1
C10B—C11B—H11A	119.9	C10A—C11A—H11B	120.1
C11B—C12B—C13B	120.2 (5)	C13A—C12A—C11A	120.1 (5)
C11B—C12B—H12A	119.9	C13A—C12A—H12B	120.0
C13B—C12B—H12A	119.9	C11A—C12A—H12B	120.0
C14B—C13B—C12B	119.6 (5)	C12A—C13A—C14A	120.8 (6)
C14B—C13B—H13A	120.2	C12A—C13A—H13B	119.6
C12B—C13B—H13A	120.2	C14A—C13A—H13B	119.6
C9B—C14B—C13B	119.5 (5)	C9A—C14A—C13A	121.8 (5)
C9B—C14B—H14A	120.3	C9A—C14A—H14B	119.1
C13B—C14B—H14A	120.3	C13A—C14A—H14B	119.1
C7B—C15B—H15A	109.5	C7A—C15A—H15E	109.5
C7B—C15B—H15B	109.5	C7A—C15A—H15F	109.5
H15A—C15B—H15B	109.5	H15E—C15A—H15F	109.5
C7B—C15B—H15C	109.5	C7A—C15A—H15G	109.5
H15A—C15B—H15C	109.5	H15E—C15A—H15G	109.5
H15B—C15B—H15C	109.5	H15F—C15A—H15G	109.5
C5B—C16B—H16A	109.5	C5A—C16A—H16E	109.5
C5B—C16B—H16B	109.5	C5A—C16A—H16F	109.5
H16A—C16B—H16B	109.5	H16E—C16A—H16F	109.5
C5B—C16B—H16C	109.5	C5A—C16A—H16G	109.5
H16A—C16B—H16C	109.5	H16E—C16A—H16G	109.5
H16B—C16B—H16C	109.5	H16F—C16A—H16G	109.5
C5B—C17B—H17A	109.5	C5A—C17A—H17E	109.5
C5B—C17B—H17B	109.5	C5A—C17A—H17F	109.5
H17A—C17B—H17B	109.5	H17E—C17A—H17F	109.5
C5B—C17B—H17C	109.5	C5A—C17A—H17D	109.5
H17A—C17B—H17C	109.5	H17E—C17A—H17D	109.5
H17B—C17B—H17C	109.5	H17F—C17A—H17D	109.5
			
C7B—N1B—C1B—C2B	−2.3 (6)	C7A—N1A—C1A—C2A	−6.1 (6)
C7B—N1B—C1B—C6B	176.5 (3)	C7A—N1A—C1A—C6A	172.2 (4)
N1B—C1B—C2B—C3B	175.0 (4)	N1A—C1A—C2A—C3A	177.8 (4)
C6B—C1B—C2B—C3B	−3.8 (6)	C6A—C1A—C2A—C3A	−0.4 (6)
C1B—C2B—C3B—O1B	−177.1 (4)	C1A—C2A—C3A—O1A	176.9 (4)
C1B—C2B—C3B—C4B	0.6 (6)	C1A—C2A—C3A—C4A	−7.3 (6)
O1B—C3B—C4B—C5B	−153.9 (4)	O1A—C3A—C4A—C5A	−149.1 (4)
C2B—C3B—C4B—C5B	28.3 (5)	C2A—C3A—C4A—C5A	35.0 (6)
C3B—C4B—C5B—C17B	70.8 (4)	C3A—C4A—C5A—C16A	68.4 (4)
C3B—C4B—C5B—C16B	−169.0 (3)	C3A—C4A—C5A—C6A	−52.3 (5)
C3B—C4B—C5B—C6B	−50.6 (4)	C3A—C4A—C5A—C17A	−172.3 (4)
N1B—C1B—C6B—C5B	159.0 (3)	N1A—C1A—C6A—C5A	161.5 (4)
C2B—C1B—C6B—C5B	−22.1 (5)	C2A—C1A—C6A—C5A	−20.2 (6)
C17B—C5B—C6B—C1B	−73.9 (4)	C16A—C5A—C6A—C1A	−75.3 (5)
C4B—C5B—C6B—C1B	47.9 (4)	C17A—C5A—C6A—C1A	164.6 (4)
C16B—C5B—C6B—C1B	166.4 (3)	C4A—C5A—C6A—C1A	44.8 (5)
C1B—N1B—C7B—C15B	81.9 (4)	C1A—N1A—C7A—C15A	82.0 (5)
C1B—N1B—C7B—C8B	−151.7 (3)	C1A—N1A—C7A—C8A	−153.6 (4)
N1B—C7B—C8B—O2B	−78.8 (4)	N1A—C7A—C8A—O2A	−71.3 (4)
C15B—C7B—C8B—O2B	46.7 (4)	C15A—C7A—C8A—O2A	53.3 (4)
N1B—C7B—C8B—C9B	160.0 (3)	N1A—C7A—C8A—C9A	167.8 (3)
C15B—C7B—C8B—C9B	−74.5 (4)	C15A—C7A—C8A—C9A	−67.6 (5)
O2B—C8B—C9B—C14B	−25.7 (5)	O2A—C8A—C9A—C10A	157.2 (4)
C7B—C8B—C9B—C14B	97.6 (4)	C7A—C8A—C9A—C10A	−80.0 (5)
O2B—C8B—C9B—C10B	157.6 (4)	O2A—C8A—C9A—C14A	−26.0 (5)
C7B—C8B—C9B—C10B	−79.2 (4)	C7A—C8A—C9A—C14A	96.9 (4)
C14B—C9B—C10B—C11B	−1.5 (7)	C14A—C9A—C10A—C11A	−1.3 (7)
C8B—C9B—C10B—C11B	175.3 (4)	C8A—C9A—C10A—C11A	175.7 (4)
C9B—C10B—C11B—C12B	0.8 (8)	C9A—C10A—C11A—C12A	−0.2 (8)
C10B—C11B—C12B—C13B	−0.2 (8)	C10A—C11A—C12A—C13A	1.6 (9)
C11B—C12B—C13B—C14B	0.5 (8)	C11A—C12A—C13A—C14A	−1.5 (9)
C10B—C9B—C14B—C13B	1.7 (6)	C10A—C9A—C14A—C13A	1.4 (7)
C8B—C9B—C14B—C13B	−175.0 (4)	C8A—C9A—C14A—C13A	−175.5 (4)
C12B—C13B—C14B—C9B	−1.2 (7)	C12A—C13A—C14A—C9A	0.0 (8)

### Hydrogen-bond geometry (Å, º)

**Table:**

*D*—H···*A*	*D*—H	H···*A*	*D*···*A*	*D*—H···*A*
N1*B*—H1*NB*···O1*B*^i^	0.82 (6)	2.12 (6)	2.874 (4)	155 (5)
O2*B*—H1*OB*···O1*B*^i^	0.96 (7)	1.75 (7)	2.701 (4)	170 (6)
N1*A*—H1*NA*···O1*A*^ii^	0.85 (6)	2.04 (6)	2.853 (4)	160 (5)
O2*A*—H1*OA*···O1*A*^ii^	0.91 (7)	1.88 (7)	2.724 (4)	155 (6)

Symmetry codes: (i) −*x*+2, *y*−1/2, −*z*; (ii) −*x*+1, *y*−1/2, −*z*+2.
